# Tracking neuroinflammation in Alzheimer’s disease: the role of positron emission tomography imaging

**DOI:** 10.1186/1742-2094-11-120

**Published:** 2014-07-08

**Authors:** Eduardo Rigon Zimmer, Antoine Leuzy, Andréa Lessa Benedet, John Breitner, Serge Gauthier, Pedro Rosa-Neto

**Affiliations:** 1 Translational Neuroimaging Laboratory (TNL), McGill Center for Studies in Aging (MCSA), Douglas Mental Health University Institute, Montreal, QC H4H 1R3, Canada; 2 Alzheimer’s Disease Research Unit, MCSA, Douglas Mental Health University Institute, Montreal, QC H4H 1R3, Canada; 3 Department of Biochemistry, Federal University of Rio Grande do Sul (UFRGS), Porto Alegre, Brazil; 4 CAPES Foundation, Ministry of Education of Brazil, Brasília, Brazil; 5 Centre for Studies on Prevention of Alzheimer's Disease, Douglas Mental Health University Institute, Montreal, QC H4H 1R3, Canada

**Keywords:** Alzheimer’s disease, Positron emission tomography, Neuroinflammation, Microglia, Astrocytes, Phospholipase A2, Amyloid-β, Hyperphosphorylated tau, Non-steroidal anti-inflammatory drugs, 18 kDa translocator protein

## Abstract

Alzheimer’s disease (AD) has been reconceptualized as a dynamic pathophysiological process, where the accumulation of amyloid-beta (Aβ) is thought to trigger a cascade of neurodegenerative events resulting in cognitive impairment and, eventually, dementia. In addition to Aβ pathology, various lines of research have implicated neuroinflammation as an important participant in AD pathophysiology. Currently, neuroinflammation can be measured *in vivo* using positron emission tomography (PET) with ligands targeting diverse biological processes such as microglial activation, reactive astrocytes and phospholipase A2 activity. In terms of therapeutic strategies, despite a strong rationale and epidemiological studies suggesting that the use of non-steroidal anti-inflammatory drugs (NSAIDs) may reduce the prevalence of AD, clinical trials conducted to date have proven inconclusive. In this respect, it has been hypothesized that NSAIDs may only prove protective if administered early on in the disease course, prior to the accumulation of significant AD pathology. In order to test various hypotheses pertaining to the exact role of neuroinflammation in AD, studies in asymptomatic carriers of mutations deterministic for early-onset familial AD may prove of use. In this respect, PET ligands for neuroinflammation may act as surrogate markers of disease progression, allowing for the development of more integrative models of AD, as well as for the measuring of target engagement in the context of clinical trials using NSAIDs. In this review, we address the biological basis of neuroinflammatory changes in AD, underscore therapeutic strategies using anti-inflammatory compounds, and shed light on the possibility of tracking neuroinflammation *in vivo* using PET imaging ligands.

## Background

Research advances over the past decade have led to the reconceptualization of Alzheimer’s disease (AD) as a progressive pathophysiological process in which the accumulation of amyloid-beta (Aβ) is thought to trigger a cascade of neurodegenerative events, including the intracellular accumulation of hyperphosphorylated tau [1,2]. From a clinical standpoint, AD is viewed as a continuum, comprising a clinically silent phase [3] (characterized by cognitive normality in the presence of AD pathology), a prodromal mild cognitive impairment (MCI) phase [4]—during which individuals exhibit cognitive dysfunction, but of insufficient severity to meet criteria for dementia—and, finally, a dementia phase [5].

In addition to the pathological hallmarks of AD, Aβ and hyperphosphorylated tau, a growing body of literature points to neuroinflammation as an important player in the pathogenesis of AD. Following a set of classic studies implicating the complement factors C1q, C4 and C3 in the formation of amyloid plaques [6,7], activated microglia and the inflammatory cytokine IL-1 were found to be elevated in AD patients [8,9]. In addition to its role in the promotion of astrogliosis [10,11], IL-1 is known to induce marked expression of the amyloid precursor protein (APP) gene [12] and α1-antichymotrypsin [13], both known components of amyloid plaques [14,15]. Further exploration of complement activation showed that while the opsonizing components were in proximity to amyloid plaques, the terminal components were associated with dystrophic neurites [16,17]. The importance of the complement system in AD was established shortly after, following the discovery that C1q possessed the ability to bind Aβ and its N-terminal fragments and thus to initiate neuroinflammation via activation of the classical complement pathway [18]. Since this early work, numerous post-mortem immunohistochemical, biochemical, and molecular studies have confirmed the presence of neuroinflammation in the brain of AD subjects (for review, see [19]).

A key issue regarding neuroinflammation in AD is whether this response is beneficial or detrimental in nature. While acute neuroinflammation seems to be an adaptive reaction aiming to restore brain integrity [20], chronic inflammation appears to be an injurious process, resulting in progressive neurodegeneration [21,22]. Clinical trials using non-steroidal anti-inflammatory drugs (NSAIDs) - initiated on the basis of numerous epidemiological studies suggesting that systemic use of NSAIDs can prevent or delay the onset of AD [23,24] - have yielded mixed or inconclusive results [25]. However, preliminary results from the Alzheimer's Disease Anti-inflammatory Prevention Trial (ADAPT) may suggest that NSAIDs can be beneficial only if administered early in the disease course, before any symptoms are evident [22]. While this *Janus face* of neuroinflammation in AD has yet to be fully understood, it is clear that neuroinflammation is an early and continuous process, present from preclinical through late stage AD [26-28].

Recently, positron emission tomography (PET) imaging agents targeting neuroinflammatory processes have been developed and offer the opportunity for non-invasive *in vivo* tracking of diverse brain inflammatory events (Table 1). Specifically, microglial activation, reactive astrocytosis and increased phospholipase activity are neuroinflammatory events amenable of quantification using PET imaging agents [29-31]. In addition to tracking the progression of AD as a function of neuroinflammatory response, the use of PET imaging agents may help shed light on the interplay between Aβ, hyperphosphorylated tau, and neuroinflammation, possibly leading to improved modeling of AD pathophysiology.

**Table 1:** Positron emission tomography imaging agents for neuroinflammation

**Process of interest**	**Biological target**	**Radiopharmaceutical**	**Reference**
Microglial activation	18-kDa translocator protein	[^11^C]PK11195	[32]
		[^11^C]AC5216	[33]
		[^11^C]PBR2806	[34]
		[^11^C]DPA-713	[35]
		[^11^C] DPA-714	[36]
		[^11^C]MBMP	[37]
		[^11^C]DAC	[38]
		[^11^C]DAA1106	[39]
		[^11^C]vinpocetine	[40]
		[^18^ F]PBR06	[41]
		[^18^ F]FEAC	[42]
		[^18^ F]FEDAC	[42]
		[^18^ F]DAA1106	[43]
Reactive astrocytes	Monoamine oxidase B	[^11^C]-DED	[30]
		[^11^C]Sch225336	[44]
Phospholipase A2 activity	Metabolism of arachidonic acid	1-[^11^C]-AA	[31]
		[^18^ F]FAA	[45]

In this review, we describe some of the most important insights provided by PET imaging agents targeting neuroinflammation in AD, revise the evidence provided by preclinical and clinical trials using NSAIDs, and underscore the role that PET biomarkers may play in terms of the development of novel therapeutic strategies, monitoring of disease progression, as well as biomarkers of target engagement.

### Imaging microglial activation using PET

Comprising approximately 10% of the cells within the central nervous system [46], microglia constitute the first line of defense against invading pathogens and other harmful agents. Under pathological conditions, microglial cells proliferate and migrate to the site of injury, acquiring phagocytic abilities and releasing various pro-inflammatory mediators [47-50]. In AD, reactive microglia in the vicinity of Aβ plaques have been repeatedly observed in both clinical [51,52] and experimental studies [53,54], with experimental models confirming Aβ-mediated release of various neurotoxic molecules by microglia [55-58]. In keeping with the biphasic hypothesis of neuroinflammation, however, additional studies have shown activated microglia to release neuroprotective cytokines such as transforming growth factor-β1, and there may be worsening of AD pathology following microglial inhibition [59].

Currently, PET imaging of microglial activation is possible using molecular agents targeting the 18 kDa translocator protein (TSPO), formerly named the peripheral benzodiazepine receptor (PBR) [60,61]. Located mainly in parenchymal glial cells, TSPO is present at low concentrations under normal physiological conditions [62], save for the ependyma, choroid plexus, and olfactory nerve layer of the olfactory bulb, which display high densities of TSPO receptors [63,64]. In response to neuroinflammation, however, TSPO levels undergo a dramatic increase, making it well-suited for assessment of microglial activation [62]. Indeed, numerous studies indicate TSPO to be a sensitive marker of reactive microglia and inflammation secondary to neurodegeneration, including of the AD type (for review see [65,66]).

Preclinical studies using PET ligands binding TSPO have been performed in transgenic (Tg) rodent models harboring human APP or tau pathogenic mutations. In the case of [^11^C]PK11195 - the prototypical TSPO ligand - the number of available binding sites (B_max_) was found to be significantly increased in the frontal cortex of AD post-mortem tissue, as compared to controls, while [^3^H](R)-PK11195 binding correlated significantly with immunohistochemically labeled activated microglia [67]. Likewise, an age-dependent increase in [^3^H](R)-PK11195 was noted in APP/PS1 Tg mice, in keeping with increased retention of [^11^C](R)-PK11195 assessed using microPET, which was again correlated with the presence of activated microglia, as determined via histopathological assessment [67]. Similar work conducted using [^11^C]AC-5216 [33], and [^18^F]FEDAA1106 [68] - TSPO ligands which are optimized for improved blood-brain barrier permeability, affinity and, in the case of [^11^C]AC-5216, kinetics - revealed increased TSPO signals in living Tg mice overexpressing human APP (APP_E6993Δ_) [69]. Importantly, the APP_E6993Δ_ model displays high levels of Aβ in the absence of fibrillary amyloid plaques [70], suggesting that amyloid dysmetabolism *per se* is sufficient to induce upregulation of TSPO-positive microglia.

**Figure 1:**
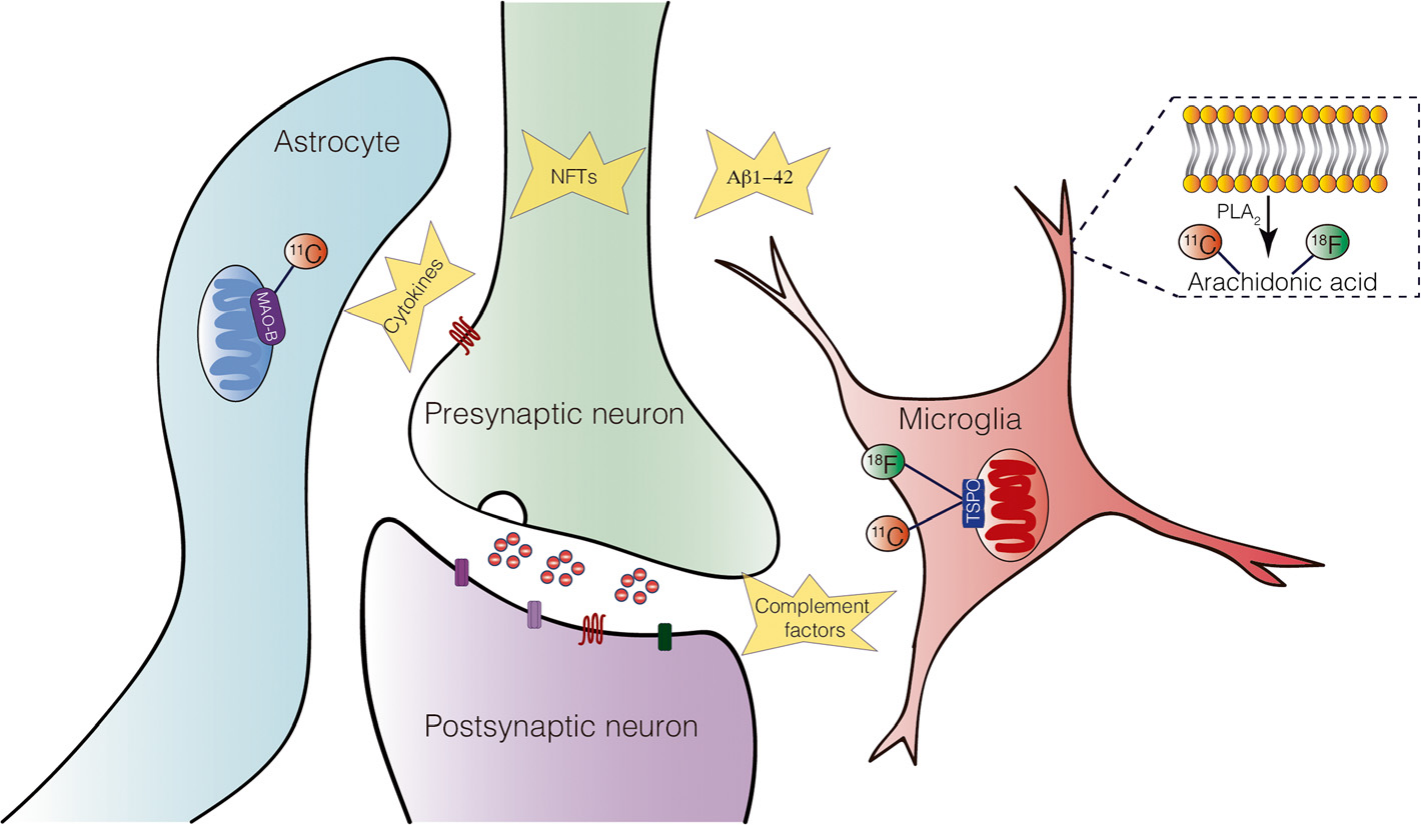
**PET biological targets for measuring neuroinflammation in AD.** Amyloid-beta (Aβ)_1–42_ and neurofibrillary tangles (NFTs) - the classic hallmarks of Alzheimer’s disease (AD) - can trigger neuroinflammatory changes, which induces the release of complement factors, cytokines and others inflammatory factors. Positron emission tomography (PET) uses biological surrogates for measuring neuroinflammation. Microglial activation is estimated by the expression of the 18-kDa translocator protein (TSPO), which is mainly found on the outer mitochondrial membrane of the microglial cells under inflammatory conditions. Monoamine oxidase-B (MAO-B), an enzyme usually located on the outer mitochondrial membrane of astrocytes, is proposed as an index of reactive astrocytosis. Radiolabeled arachidonic acid (AA), a phospholipid present in the cell membrane and cleaved by phospholipase A2 (PLA_2_), can estimate the AA metabolism. AA is the precursor of eicosanoids - prostaglandins and leukotrienes - which are potent mediators of the inflammatory response.

Clinical studies using [^11^C](R)-PK11195 in patients with mild-to-moderate AD have shown increased retention in the entorhinal, temporoparietal and posterior cingulate cortices, areas that show decreased glucose use, as measured with [^18^F]DG-PET [71]. Furthermore, elevated microglial activation, as indexed by high [^11^C](R)-PK11195 binding within cortical association and striatal regions (see Figure 1), was noted in a group of AD subjects with high Pittsburgh compound B ([^11^C]PIB) retention, whose mini mental state examination (MMSE) scores were correlated negatively with microglial activation, but not with [^11^C]PIB binding [29]. Additional studies have provided conflicting results [72]; however, it is possible that [^11^C](R)-PK11195 sensitivity might be insufficient for detecting microglial activation present in mild-to-moderate AD [73]. In MCI, findings with [^11^C](R)-PK11195 are inconclusive, with one study showing a small increase in PIB-positive patients relative to controls [74], but others studies reporting no increase, even among patients who subsequently converted to AD [72,73].

**Figure 2:**
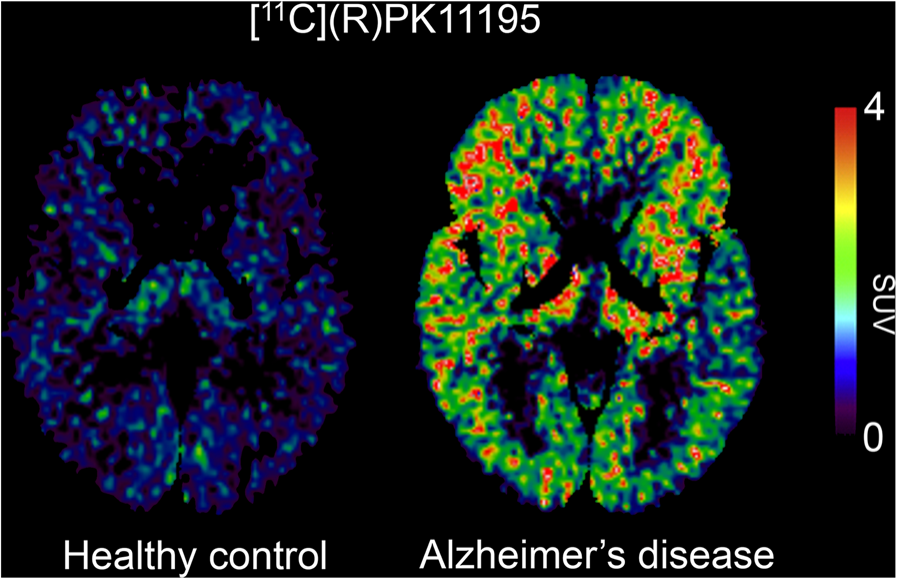
**Illustrative [**^**11**^**C](R)PK11195 PET imaging.** Representative [^11^C](R)PK11195 images in a healthy control (age 65 years) and in a patient with Alzheimer’s disease (AD) dementia (age 68 years). The brain axial view shows increased [^11^C](R)PK11195 binding in the AD subject (yellow-red spots) in comparison to the healthy control subject. Standardized uptake value (SUV) defined by the ratio of brain to reference region (supervised reference tissue extraction) radioactivity was used for estimating [^11^C](R)PK11195 binding. Image provided by Dr Paul Edison of the Division of Brain Sciences, Department of Medicine, Imperial College London, UK.

Among second generation TSPO radioligands, increased binding of [^11^C]DAA1106 has been observed in AD, as compared to controls, though no correlation was found with respect to disease severity [75]. In a follow-up study among patients with MCI, the increased binding was associated with progression to AD over a 5-year follow-up period [39]. In the case of [^11^C]PBR28, increased binding was noted in AD, but not MCI, despite the latter displaying cerebral amyloidosis and hippocampal atrophy using PIB-PET and magnetic resonance imaging (MRI) volumetry [76]. Furthermore, [^11^C]PBR28 binding was shown to correlate with clinical severity and gray matter loss, particularly within regions exhibiting the highest density of TSPO [76] (see Figure 2). Finally, [^11^C]PBR28 binding was found to be higher among patients with early-onset AD (<65 years), particularly within frontal and parietal regions, in keeping with studies showing greater frontoparietal atrophy in patients with early-onset AD [77-80]. Collectively, these findings with [^11^C]PBR28 suggest that the increased expression of TSPO by activated microglia occurs after progression to AD, and continues as a function of disease progression, in those who develop disease symptoms at an early age.

### Second-generation 18-kDa translocator protein ligands and the rs6971 polymorphism

**Figure 3:**
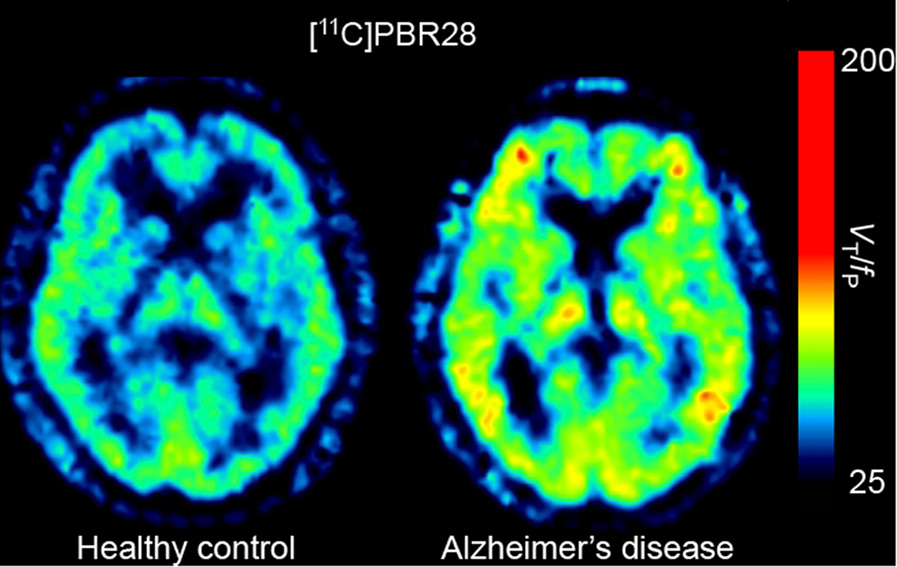
**Illustrative [**^**11**^**C]PBR28 PET imaging.** Representative [^11^C]PBR28 images in a healthy control (age 61 years) and in a patient with Alzheimer’s disease (AD) dementia (age 57 years). The brain axial view show increased [^11^C]PBR28 binding in the AD subject (yellow-red spots) in comparison to the healthy control subject. Of note, both subjects are high-affinity binders. Distribution volume corrected for free fraction of the radioligand in plasma (*V*_*T*_/*f*_P_) was used for estimating [^11^C]PBR28 binding values. Image provided by Drs William Kreisl and Robert Innis of the Molecular Imaging Branch, National Institute of Mental Health-NIMH, USA.

In contrast to PET studies using [^11^C]PK11195 - which binds with similar affinity across subjects [81] - interpretation of studies using second-generation ligands for the TSPO has been rendered difficult by substantial inter-individual variability in binding affinity, ranging from 4- to 50-fold [82]. Recently, a common single-nucleotide polymorphism (rs6971) in exon 4 of the TSPO gene has been identified as the key determinant of TSPO ligand affinity [83,84]. Based on the rs6971 polymorphism, subjects are currently divided in three groups: high-affinity binders (HAb), mixed-affinity binders (MAb) and low-affinity binders (LAb), with binding class determined on the basis of the number of high- versus low-affinity sites [81,82]. While the binding variation between HAb and MAb is around 30% [85], a difference approaching 80% has been observed between between HAb and LAb [76]. Adjusting for the rs6971 polymorphism - either via genotyping or by leucocyte binding assay - has been shown to result in more accurate quantitation of TSPO availability [76], as well as offering potential benefits of increased statistical power and smaller required sample size in the case of clinical studies (see Figure 3).

### Cannabinoid receptor type 2: a potential target for imaging microglial activation using PET

Under physiological conditions, the cannabinoid receptor type 2 (CB_2_) is expressed in very low concentrations in the brain [86,87]. Recent studies, however, have demonstrated microglial overexpression of CB_2_ in AD following Aβ deposition [88,89]. An attractive alternative to TSPO ligands, CB_2_ radiopharmaceuticals, such as [^11^C]Sch225336 [44] and [^11^C]A-836339 [90], are potential imaging biomarkers for estimating activated microglia in the brain. Indeed, work using [^11^C]A-836339 has provided the first *in vivo* evidence of CB_2_ upregulation in APPswe/PS1dE9 mice [90], an animal model presenting Aβ deposition similar to that seen in AD. Moreover, preliminary findings highlight the CB_2_ receptor as a potential therapeutic target, with use of selective CB_2_ agonist shown to reduce microgliosis, promote Aβ clearance, and improve cognitive performance in both Tg APP 2576 mice [91] and in rats with cognitive impairment following bilateral microinjections of Aβ at the level of the hippocampus [92]. In addition, Tg amyloid mice lacking the CB_2_ receptor have been shown to exhibit significantly increased levels of soluble Aβ_1–42_ and plaque deposition [93]. Though the use CB_2_ imaging agents may play a role in monitoring the effectiveness of CB_2_-specific interventions, more studies are needed. The link between TSPO and CB_2_ expression, however, remains elusive.

### Imaging reactive astrocytes using PET

Astrocytes are the most prevalent cells in the central nervous system, outnumbering neurons by at least five-fold [94]. These specialized glial cells dynamically interact with neurons modulating diverse signaling pathways as well as synapse formation [95-97]. Similar to microglia, astrocytes become reactive in response to a variety of detrimental stimuli [98]. In AD, increased expression of glial fibrillary acidic protein (GFAP) is typically observed in immunohistochemical studies of post-mortem brain tissue, indicating an increased number of reactive astrocytes [99], with GFAP-positive astrocytes noted at the margins of amyloid deposits [100]. Though the astrocytic network is thought to exert a neuroprotective role via the sequestration/degradation of Aβ [101-105], its involvement is likewise believed to extend in deleterious directions, including the amplification of cortical amyloid deposition via propagation of intercellular calcium waves [106].

In PET imaging, the enzyme monoamine oxidase B (MAO-B) has been proposed as a biomarker for *in vivo* quantification of astrocytosis in AD [107]. Located on the outer mitochondrial membrane, MAO-B occurs predominantly in astrocytes [108,109], and can be imaged using 11C-deuterium-L-deprenyl ([^11^C]-DED), a radiopharmaceutical exhibiting high affinity and specificity for MAO-B [110,111]. Catalyzing the oxidative deamination of catecholamines, MAO-B density has been shown to be highly expressed in astrocytes surrounding amyloid plaques [112] and seems to contribute to neurodegeneration by disrupting oxidative homeostasis [113].

Clinical studies using [^11^C]DED have demonstrated increased tracer retention in MCI and AD, with binding highest among PIB + MCI individuals [30]. These findings suggest that reactive astrocytosis may be present early on in the course of AD, in keeping with previous hypotheses [99,114], as well as with findings of increased [^11^C]DED binding in the earliest Braak stages of AD post-mortem tissue [115]. Several important factors remain unanswered with respect to this study, however. First, [^11^C]DED binding may be underestimated since it is highly dependent on cerebral blood flow [111,116,117], a parameter known to be reduced in AD [118-121]. Moreover, it is unclear the degree to which the [^11^C]DED signal represents reactive astrocytosis, since MAO-B is also found within serotonergic neurons and non-reactive astrocytes [108,109]. Another confounder that needs to be addressed is the fact that MAO-B seems to increase with age in almost all brain regions (with the exception of the cingulate gyrus) in healthy human subjects [111].

### Imaging phospholipase A2 using PET

Microglia derived inflammatory cytokines are capable of binding astrocytic cytokine receptors that are coupled to cytosolic phospholipase A2 (cPLA_2_), and secretory phospholipase A2 (sPLA_2_) [122]. Activation of these Ca^2+^-dependent enzymes results in the hydrolysis of membrane phospholipids, liberating arachidonic acid (AA) [123,124], itself a precursor of pro-inflammatory eicosanoids including prostaglandins and leukotrienes [125,126]. Moreover, nitric oxide, released as part of this reaction, can likewise promote AA hydrolysis via cPLA_2_ via postsynaptic ionotropic N-methyl-D-aspartate glutamate receptors [127,128], as can Aβ [129,130]. Such mechanisms have been noted in AD, including increased expression of cPLA_2_ and sPLA_2_, elevated cytokine levels, increased glutamatergic markers and different forms of accumulated Aβ [129-131], as well as increased cerebrospinal fluid (CSF) levels of AA metabolites [132]. On the basis of these markers, AA metabolism has been hypothesized to be elevated in AD [31].

Preliminary results obtained using radiolabeled AA (1-[^11^C]-AA) support this hypothesis, with elevated incorporation coefficients noted in neocortical areas shown to have high densities of neuritic plaques with activated microglia [31]. To the extent that the elevated binding of 1-[^11^C]-AA represents the upregulation of AA metabolism, PET with 1-[^11^C]-AA may prove of use in the assessment of investigations in patients with AD.

### Neuroinflammation as a therapeutic target in AD

Following an initial report of unexpectedly low prevalence of AD among patients with rheumatoid arthritis [133], numerous epidemiological studies have indicated a reduced incidence of AD among users of NSAIDs (for a review, see [134]). A systematic review suggests that duration of NSAID use of at least 2 years is required to reduce risk estimates [135], while the apparent protective effects of NSAIDs is diminished among older individuals, even to the point of disappearance [136,137] or, in one study, reversal [138]. The apparent protective effect may also be more pronounced among carriers of the apolipoprotein E (*APOE*) ϵ4 allele [24,137,139,140]. In general, non-aspirin compounds have been associated with greater protective effects, relative to aspirin compounds [134], and no protective effects have been suggested among users of acetaminophen [141].

Several mechanisms have been proposed to explain the possible protective effects of NSAIDs in AD, including the reduction of brain inflammation by inhibition of cyclooxygenase (COX)-mediated synthesis of pro-inflammatory prostaglandins [101]. Indeed, the beneficial effects of NSAIDs on memory performance in transgenic mouse models of AD have been proposed to relate directly to their blockade of COX activity [102-105,142,143], and not to their ability to lower levels of inflammatory cytokines, TNF-α or IL-1β [144]. A second proposed mechanistic hypothesis pertains to the ability of NSAIDs to inhibit processing of the APP or the production or aggregation of Aβ. A subset of NSAIDs (including ibuprofen and indomethacin) have been shown *in vitro* and in APP-Tg mouse models to preferentially lower levels of amyloidogenic Aβ_1–42_, independently of their COX-inhibiting activity [145]. These NSAIDs are thought to stimulate non-amyloidogenic processing of APP via enhancement of α-secretase activity [146], to decrease secretion of Aβ in cell lines stimulated with pro-inflammatory cytokines [147], and to decrease the expression of α1-antichymotrypsin, an acute phase protein known to accelerate the development of amyloid pathology in APP-Tg mice [148]. It is noteworthy, however, that a large meta-analysis of NSAID use in human samples has failed to show a distinction between those NSAIDs that modulate γ-secretase activity *in v*itro and others, and between ibuprofen and naproxen in particular [149]. Finally, NSAIDs that are known to inhibit the multimerization of Aβ *in vitro*[150] may also inhibit the aggregation of Aβ via direct interaction [151], although human neuropathological studies have failed to show this [152,153].

The hypothesis that neuroinflammation plays a role in the pathogenesis of AD - and that its suppression via the use of anti-inflammatory compounds may prevent or delay the onset of AD - provided the rationale for a series of clinical trials utilizing various anti-inflammatory drugs [154]. While initial pilot studies using indomethacin (a COX-1 preferential inhibitor) and diclofenac (a non-selective COX inhibitor) combined with the gastroprotective agent misoprostol, suggested benefits in mild-to-moderate AD [155,156], gastrointestinal problems (a side effect commonly associated with inhibition of COX1) resulted in a high drop-out rate. Follow-up studies with nimesulide [157], celecoxib [158] or rofecoxib [159,160] - selective COX-2 inhibitors - showed no therapeutic effect [157-160]. Likewise, despite encouraging results in animal models, a 1-year clinical trial using the non-selective COX inhibitor ibuprofen showed no significant overall effects on cognitive and clinical outcomes in patients with mild-to-moderate AD (although positive results were seen in *APOE* ϵ4 carriers, with the opposite pattern observed in non-carriers) [161]. A primary prevention trial (ADAPT) using naproxen (mixed COX-1/COX-2 inhibitor) and celecoxib (selective COX-2 inhibitor) in individuals at risk for AD was terminated early due to perceived cardiovascular side effects [162], but with results that suggested that these NSAIDs may in fact accelerate disease progression if initiated in individuals already displaying MCI or substantial AD pathophysiology without symptoms. Their use among individuals without AD pathophysiology may prove beneficial, however; a hypothesis supported by CSF biomarker values obtained at 21 to 42 months follow-up [134].

Though the failure of clinical trials using NSAIDs has been ascribed to timing of intervention, duration of treatment, dosage, and drug class, the underlying problem remains the lack of consensus surrounding whether neuroinflammation causes neurodegeneration in AD, or is simply a protective response to primary pathological processes. At present, these findings offer only limited data making it difficult to evaluate the therapeutic utility of NSAIDs in AD. However, piecing together findings from preclinical work in AD-like Tg models, the finding of protective effects in *APOE* ϵ4 carriers and the overall results of the ADAPT trial, it seems likely that NSAIDs may exert a protective effect but only if administered early on in the disease course. Further studies are required to provide support for this idea.

### Potential role of PET in monitoring responsivity to anti-inflammatory therapies in AD

The literature suggests that neuroinflammation occurs early in the course of AD, likely as a response to Aβ and pathologically phosphorylated forms of tau, and that early use of NSAIDs may prove effective in individuals with minimal AD pathology and/or carriers of the *APOE* ϵ4 allele. Indeed, results from a masked long-term follow-up of the ADAPT cohort seemed to confirm this idea. The difficulty with early initiation of treatment, however, lies in recognizing those who have already experienced pre-symptomatic disease onset. In this respect, studies in asymptomatic carriers of mutations deterministic for early-onset familial AD may prove helpful, given the recent suggestion of the order in which biomarkers reach abnormal levels in this population [163], and that the clinical evolution of early-onset familial AD is highly predictable within a kindred, especially for age at onset [164]. Imaging of neuroinflammation in this population using PET, alongside CSF and plasma markers, may allow for a more integrative AD biomarker model [2,165], particularly with respect to the interplay between glial activation, seeding of Aβ and hyperphosphorylated tau species, and cognitive decline. Moreover, TSPO ligands may prove sensitive to early AD pathophysiological changes in the form of toxic Aβ oligomers [166], changes that lie below the detection threshold of current *in vivo* AD biomarkers [2]. In the context of clinical trials using NSAIDs (compounds that retain therapeutic potential despite the general failure of trials conducted to date, particularly since they are relatively safe and highly available), PET could serve to demonstrate target engagement in addition to proving topographical information, critical information lacking from fluid- and plasma-based biomarkers. The reproducibility of TSPO binding, however, remains under debate [167,168]. In this respect, clinical trials addressing this issue are currently underway, and will hopefully provide results clarifying the test-retest reliability of TSPO ligands [169,170].

### Concluding remarks and future directions

In general, the continued study of inflammatory mechanisms, including in particular the use of PET imaging for tracking neuroinflammatory changes, seems to have a promising role in AD. To date, radioisotopic probes targeting neuroinflammation have demonstrated encouraging results in preclinical and clinical studies, with these radiopharmaceuticals holding promise for inclusion as surrogate markers of disease progression in the next generation of clinical trials using anti-inflammatory therapies. Importantly, novel approaches aiming to augment the sensitivity of these PET imaging agents may be required, with inclusion of vascular [171] and genetic [172,173] covariates likely to strengthen the value of PET outcomes.

## Abbreviations

[11C]-DED: 11C-deuterium-L-deprenyl; [11C]PIB: Pittsburgh compound B; Aβ: amyloid-beta; AA: arachidonic acid; AD: Alzheimer’s disease; ADAPT: Alzheimer's Disease Anti-inflammatory Prevention Trial; APOE: apolipoprotein E; APP: amyloid precursor protein; CB2: cannabinoid receptor type 2; COX: cyclooxygenase; cPLA2: cytosolic phospholipase A2; CSF: cerebrospinal fluid; GFAP: glial fibrillary acidic protein; HAb: high-affinity binders; IL: interleukin; LAb: low-affinity binders; MAb: mixed-affinity binders; MAO-B: monoamine oxidase B; MCI: mild cognitive impairment; NSAID: non-steroidal anti-inflammatory drug; PBR: peripheral benzodiazepine receptor; PET: positron emission tomography; sPLA2: secretory phospholipase A2; Tg: transgenic; TNF: tumor necrosis factor; TSPO: 18 kDa translocator protein.

## Competing interests

The authors declare that they have no competing interests.

## Authors’ contributions

ERZ and AL were responsible for the conception and design of the review, and for drafting and revising the manuscript. ALB, JB, SG and PRN were responsible for revising the manuscript. All authors read and approved the final manuscript.
